# The sydney playground project: popping the bubblewrap - unleashing the power of play: a cluster randomized controlled trial of a primary school playground-based intervention aiming to increase children's physical activity and social skills

**DOI:** 10.1186/1471-2458-11-680

**Published:** 2011-09-01

**Authors:** Anita C Bundy, Geraldine Naughton, Paul Tranter, Shirley Wyver, Louise Baur, Wendy Schiller, Adrian Bauman, Lina Engelen, Jo Ragen, Tim Luckett, Anita Niehues, Gabrielle Stewart, Glenda Jessup, Jennie Brentnall

**Affiliations:** 1 Faculty of Health Sciences, University of Sydney, Lidcombe, Australia; 2 Centre of Physical Activity across the Lifespan, Australian Catholic University, Melbourne, Australia; 3 School of Physical, Environmental and Mathematical Sciences, University of New South Wales, Canberra, Australia; 4 Institute of early childhood, Macquarie University, Sydney, Australia; 5 Paediatrics & Child Health, University of Sydney, Westmead, Australia; 6 School of Education, University of South Australia, Adelaide, Australia; 7 Public Health, University of Sydney, Sydney, Australia

## Abstract

**Background:**

In the Westernised world, numerous children are overweight and have problems with bullying and mental health. One of the underlying causes for all three is postulated to be a decrease in outdoor free play. The aim of the Sydney Playground Project is to demonstrate the effectiveness of two simple interventions aimed to increase children's physical activity and social skills.

**Methods/Design:**

This study protocol describes the design of a 3-year cluster randomised controlled trial (CRCT), in which schools are the clusters. The study consists of a 13-week intervention and 1 week each of pre-and post-testing. We are recruiting 12 schools (6 control; 6 intervention), with 18 randomly chosen participants aged 5 to 7 years in each school. The two intervention strategies are: (1) Child-based intervention: Unstructured materials with no obvious play value introduced to the playground; and (2) Adult-based intervention: Risk reframing sessions held with parents and teachers with the aim of exploring the benefits of allowing children to engage in activities with uncertain outcomes. The primary outcome of the study, physical activity as measured by accelerometer counts, is assessed at baseline and post-intervention. Additional assessments include social skills and interactions, self-concept, after school time use and anthropometric data. Qualitative data (i.e., transcriptions of audio recordings from the risk reframing sessions and of interviews with selected teacher and parent volunteers) are analysed to understand their perceptions of risk in play. The control schools have recess as usual. In addition to outcome evaluation, regular process evaluation sessions are held to monitor fidelity to the treatment.

**Discussion:**

These simple interventions, which could be adopted in every primary school, have the potential of initiating a self-sustaining cycle of prevention for childhood obesity, bullying and mental ill health.

**Trial registration:**

Australian and New Zealand Clinical Trials Registration Number ACTRN12611000089932.

## Background

Disturbing numbers of children in the westernised world are overweight, bullied or have poor mental health. These are all serious childhood problems that are notoriously difficult to treat. Prevention is undoubtedly best, but their continued increase shows how difficult it is to reverse these new and dangerous trends. Burdette and Whitaker [1] suggested a compelling common cause for all three problems: a decrease in outdoor free play. While previous generations spent most of their discretionary time engaged in free play outdoors, today's children play indoors; their free time is often spent in sedentary and solitary activity (e.g., computers, television). Time spent outdoors is often in structured sport rather than in unstructured play that facilitates social interaction and physical activity. Physical activity promotes energy expenditure and has additional benefits such as improved cardiovascular health and gross motor skills. However, less obvious is the role of free play in children's social, emotional and cognitive development. The shift from outdoor play to indoor activities, combined with an increased push for academic achievements, potentially leave children more stressed and with poorer mental health [1]. Most young children love active outdoor play but parents, teachers and carers may limit this, fearing injury or misadventure.

### The issues

#### Risk aversion in the short term can lead to greater risks in the long term

Adult fears are often disproportionate to actual risk: while children can be injured when playing outdoors, most are minor cuts and bruises. On the other hand, restricting children's outdoor play activities may have unintended consequences, such as reducing children's opportunities for reasonable, age-appropriate risk-taking. Fear of litigation results in minimising "risk" at all cost and decreasing the value of "real play" that occurs on school playgrounds [2,3]. Several schools have now placed a ban on running on the school playground [4,5]. Thus, "surplus safety" may result in exactly the negative outcomes it was meant to avert [6]. Although no research has directly examined risk avoidance and children's health outcomes, recent Australian data suggest that concern with children's risk exposure can lead to constraints on children's habitual physical activity, which may have negative public health outcomes [7]. Further, when children perceive that play settings are not demanding enough, they may compensate by engaging in activities that yield challenges -- in the context of undesirable behaviour (e.g., bullying or using play equipment in truly dangerous ways)[8].

#### Social and Physical Environments Affect Play

Opportunities to engage in spontaneous play in the local neighbourhood have declined in recent generations [9]. The age at which children are allowed outside to play on their own has increased in recent years [10]. While parents of older children may have greater tolerance for allowing their children to take manageable risks, young children are generally not allowed to engage in what parents perceive as risky play or activities. Mullins and colleagues [11] speculated that parents are less likely to engage in overt overprotective behaviours as their children reach adolescence, yet still perceive their child as vulnerable. Children are often driven by car to play activities [12] and these activities are likely to be adult-organised and indoors [13]. When children are allowed to play outside, it is often on the school playground for which rules limit play behaviours [14] and purpose-built equipment (e.g., slide) fails to promote collaborative and imaginative play. Slides and climbers are also the source of most serious playground injuries [15] and climbers are often places in which less physically competent children are teased by peers [16].

In contrast, natural play spaces (e.g., woods, gardens) and playgrounds with unstructured construction materials stimulate diverse and creative play [17]. Fantasy and socialisation are prominent and the social hierarchy is based on the ability to imagine what the space might become rather than on physical prowess [18]. Children experience active play through lifting, pushing, and pulling. They engage in creative, socially interactive play as they construct new structures and play within them [19]. Both in our pilot study [20,21], and in a study at another Australian school where children played with unstructured materials, reports of injuries, bullying and fighting were almost non-existent [14].

#### Sedentary lifestyle and obesity

In Australia over 200,000 children are obese [22]. Childhood obesity is a serious problem with a range of significant medical, psychosocial and economic consequences [23]. Despite the growing awareness of the consequences, the prevalence of childhood overweight and obesity is still high in many westernised and westernising countries [24,25]. Regular physical activity is associated with both a longer and healthier life [26], including a decreased risk of obesity [27], insulin resistance [28] and metabolic risk [29]. Similarly, physical *inactivity *during the early years of life is a major contributor to serious medical conditions in children [30,31]. Time spent participating in sedentary and low energy-expending activities is associated with higher relative weight [32-34] and poorer health. Hence, a reduction in sedentary behaviour is important for promoting health [35].

Several underlying issues may contribute to the increasingly sedentary lifestyles of some children. In many cases children are reliant on parents when it comes to the activities they undertake. Families live exceedingly busy lives with limited time for children's free play and unstructured activity. To "make the most" of the time they have, many middle-class parents enrol children in organised activity and sport [36,37] that offer strictly scheduled, structured exercise and play. In families with fewer financial resources, children are more likely to spend more time in front of the television instead [36]. To make sure children's school results do not suffer, there is an increased emphasis on academic tasks, particularly in middle and upper class families. This increased pressure on children, in combination with the perception that there are many "predators" in public spaces, ensures an increase in screen- and homework time and a decrease in time spent outdoors. To save time and keep children "safe," many parents drive them to school and sport--creating a self perpetuating cycle.

#### Mental ill health and bullying

About 14% of Australian children have significant mental health problems [38]. In children from disadvantaged backgrounds, this figure can rise up to 60% [39]. Play is one of the best ways for children to develop coping skills, and thereby mental health. Play engages children's intrinsic motivation and supports autonomy, competence and relatedness that have been shown to promote psychological wellbeing [40].

Poor social skills have been linked both to bullying and being bullied [41] and children with higher self-concept are bullied less often and also are actively included in play more often [42,43]. Our conjecture is that unstructured materials on the playground will give children who are not very sporty or physically coordinated the chance to play. Teachers participating in our pilot study reported that the unstructured materials promoted play between children who had not played together previously--including children who had formerly been excluded [44].

#### Play as an alternative intervention

Play is a universal and profound process that has evolved in both animals and humans. It is spontaneous, exploratory and intrinsically motivated [45]. Children love to explore and delight in opportunities to devise their own free play to experience sheer joy and pleasure in the moment [46]. There are many documented benefits to play [47] and this study aims to engage children's playful nature to promote greater physical activity and to enhance social skill development. When children play freely with materials that are intriguing and that also require physical exertion, we believe that they will spend more time engaging in the activity and thus also be more active for longer periods of time. When materials are large, a little unwieldy, or a bit heavy, it is possible that, in addition to expending more energy, and exerting greater effort, they will recruit help from other children, thus promoting social negotiation in the context of creative play (i.e., what can *we *do with these materials?). Play also offers opportunities for children to take manageable risks and to experience the benefits of a variety of challenges (e.g., motor, social, emotional and cognitive). The playground based intervention presented here is aimed at appealing to children's inherent will to play, rather than imposing a structured program or regimen.

Prevention strategies that target the population as a whole, rather than focussing only on children at risk are preferable. It is difficult to identify who is at risk and all children benefit from increased activity independent of body composition and interest [48]. Thus programs conducted at schools can be highly beneficial.

Previous studies have supported play and changes to the playground as a way of increasing activity. In the UK, painting lines on the playground to promote active games resulted in small but significant and lasting increases in children's physical activity [49-51]. Placing play equipment and activity cards on school playgrounds in Belgium significantly increased the children's moderate to vigorous physical activity (MVPA) [52]. Similar results were found in North Carolina where portable play equipment, including skipping ropes and hula hoops, at day care facilities increased physical activity [53]. Turning a Canadian school playground into a natural play space also increased physical activity significantly [54]. The same group of researchers also found that a higher percentage of children were engaged in MVPA on green areas and on fixed playground equipment than on sporting courts and other courtyards [55].

A few previous researchers have introduced unstructured materials to school playgrounds and studied the effect on social interaction and play. One of them is a Play Pods study, carried out in Bristol from 2006 to 2009 [56]. The overall impression of the outcomes, determined by observations and interviews with children and school staff, was very positive. The researchers observed that the loose items improved children's participation, decision making skills and their control of play within school setting, as well as improved access for all children to inclusive play opportunities. We build on studies such as this with quantitative data.

## Aims and hypotheses

The aim of the Sydney Playground Project is to demonstrate the effectiveness of a simple, cost-effective two-part intervention to increase children's physical activity, social skills and resilience by altering their experience on the school playground. The two intervention strategies are:

a. Child-based intervention: Recycled unstructured materials are placed on the school playground with the aim of increasing physical activity and social skills through unstructured free play.

b. Adult-based intervention: Risk reframing sessions held with parents and teachers with the aim of exploring the benefits of allowing children to engage in activities with uncertain outcomes.

## Methods/Design

### Overall study design

**Figure 1:**
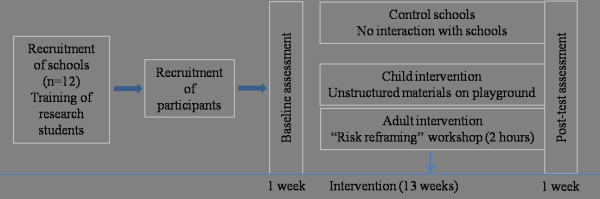
**Study design of Sydney Playground Project**.

The design of this 3-year study is a cluster randomised controlled trial (CRCT) in which the participating schools are the clusters. The study consists of a 13-week intervention with baseline and post-testing (See Figure 1). The study has been approved by the Human Research Ethics Committee at the University of Sydney and by the Catholic Education Office of the Archdiocese of Sydney.

### Participants and recruitment

Catholic co-educational primary schools are approached to participate in the study, through emails, phone calls and on-site visits to the principal and/or assistant principal. The schools are located within a 30 km radius of the University of Sydney's Health Sciences campus at Lidcombe, near Sydney. Recruitment continues until 12 schools agree to participate.

### School selection

All participating schools must agree to be randomised into the control or intervention group and refrain from engaging in other new interventions designed to increase activity levels. They continue with normal recess and physical education. There are no exclusion criteria.

### Child selection

Within each school, a random sample of children is invited to participate. Children are selected using the following procedure: To maintain confidentiality, schools assign each of their Kindergarten and Year 1 students (aged 5 to 7 years) a number. Schools are then presented with a set of randomly generated numbers. School staff approach children assigned to the random numbers until 20 students and their parents consent to participate. Data are gathered on country of birth of children and parents as well as languages spoken. Families are approached only if the parents can speak and understand spoken and written English to a degree sufficient to allow them to complete the questionnaires and participate in the risk reframing intervention. Children are included regardless of known disability.

### Sample size

Sample size calculation involves deciding on both the number of schools (clusters) and number of children per school. The desired number of schools provides sufficient cluster-level data for subsequent analyses but, at the same time, is manageable in terms of implementation and data collection. We have selected 12 schools (6 control, 6 intervention) to meet these criteria.

The number of children needed per school is estimated by considering the amount of change from the intervention and correcting for the possibility that children at the same school affect one another's activity levels (determined by intra-cluster correlation [ICC]) We estimated the effect size from the intervention conservatively at 0.5 standard deviations (SD) based on a report that young children engage in moderate to vigorous playground activity 26% of the time and that 40% would be a realistic health-promoting target [57].

With *p *= 0.05, power = 80% and an ICC of .04, we require 18 children per school to show that an effect size of 0.5 SD is significant between groups. This reflects a 68% inflation in sample size over the number that would be required if no adjustment were made for cluster effect (64 vs. 108 participants/group).

### Randomisation

The 12 participating schools are randomly assigned to either the control or intervention cluster. Randomisation is performed prior to the commencement of the study by a researcher unrelated to the study; researchers are blinded to school allocation until after baseline data collection at the relevant school. The schools are informed of their allocated treatment after baseline data collection.

### Interventions

#### Child-based intervention

Unstructured materials are introduced on the school playgrounds to be used during recess. The materials are selected to conform to seven principles: 1. no obvious play value; 2. encourage cooperative, gross motor play; 3. have multiple uses; 4. can be used in challenging, creative and uncertain ways; 5. provide interesting sensory experiences (e.g., from touch or movement); 6. inherent hazards can easily be seen/managed by a child; 7. are, or are made from, re-cycled materials. Examples of items include car tyres, milk crates, and cardboard boxes; in some cases these are reinforced or made more water resistant (e.g., by drilling tyres or covering cardboard with plastic tape). Some items, such as crash mats, are fabricated from recycled objects. All materials are checked for safety characteristics and child-proofed; all meet Australian standards for playground materials. New materials are introduced periodically to replace broken objects or to complement existing objects. Maintenance of the materials is the responsibility of researchers in collaboration with each school community but school staff must agree to remove materials that are broken or being used in unsafe ways.

This intervention runs in each intervention school over 2 terms (13 weeks total), during which time children are able to use and play freely with the items. The materials are for the use of all children at the school, independent of project consent.

#### Adult intervention: Risk Reframing

Teachers and parents of participating children from the intervention schools are asked to participate in a 2-hour group intervention. The adults participate in small "task groups" (*n *= 6-8) as well as large whole-group discussions (*N *= 18-24) to examine their own experiences of free play and their beliefs regarding the benefits and risks associated with active play. Discussions focus on similarities and differences of parents' and teachers' perceptions of the benefits of play, healthy (or manageable) vs. unhealthy risk and the consequences of preventing children from engaging in play and healthy risk taking. Schools are offered compensation for the time the teachers are participating in the sessions.

### Control schools

The control schools participate in standard recess, and they do not have access to the unstructured materials or to risk reframing.

### Outcome measures

The primary outcome measure is children's physical activity, as measured by accelerometers. The secondary outcomes include: self-concept and social skills, social interactions, after school time use and anthropometric measures. All the measurements are performed during one full school week (Table 1).

**Table 1:** Outcome measures and schedule of the study

Outcome	Measure	Data collection	Dependent variable	Baseline	Post-test
BMI	*Stadiometer and scales*	Researcher with child	Height and weight	1 occasion	1 occasion
Physical Activity	*Actigraph accelerometer^1^*	Devices worn by children	Counts, and minutes in sedentary and moderate to vigorous activity	5 days	5 days
	*Activity Diary^2^*	Palm pilots responded on by parents	Activities undertaken after school	4 days	4 days
Social Interaction	*Playground Behaviour*	Video recording	Social behaviour	15 min	15 min
	*SSIS-RS ^3^*	Parent and teacher reports	Social skills and problem behaviour	1 occasion	1 occasion
Self-concept	*PSPCSAYC^4^*	Researcher with child, and teacher reports	Feelings of competence	1 occasion	1 occasion

^1 ^www.theactigraph.com

^2 ^Palm pilot Z22

^3 ^Social Skills Improvement System Rating Scales [61]

^4 ^Pictorial Scale of Perceived Competence and Social Acceptance for Young Children [60]

### Measurements

#### Physical activity during school days

Actigraph accelerometers (GT3X, http://www.theactigraph.com) are worn on the participating children's right hip, on the iliac bone, on top of clothing and fastened with an elastic waist band. A researcher attaches the accelerometers at 9.00 AM and removes them at 3.00 PM for 5 consecutive school days. Movement is measured in three planes at 5 second epochs.

Data are downloaded from the accelerometers using the product-coupled software ActiLife and stored as .dat files to be used in subsequent analyses. To measure the level of physical activity, total accelerometer counts, as well as minutes and percent of time spent in sedentary, light, moderate and vigorous physical activities are calculated, using a custom-made macro (Stewart Trost). The cut-points for levels of physical defined by Evenson et al. [58] are used. Accelerometer non-wear time is defined as a count of 0 for 10 consecutive minutes [59].

#### Children's activities after school

We collect information about activities during after-school hours (between 3.30 PM and 7.00 PM) in both baseline and post-test sessions using a real-time activity diary. Responses to the activity diary are recorded at three random times for each of 4 consecutive week days. The activity diary consists of 12 questions (9 multiple choice, 3 Visual Analogue Scales [VAS]). The multiple choice questions determine who is recording the information, what the child is doing and using, who the child is with, if the television is on and whether they are indoors or outdoors. The VAS questions are used to estimate, 1) activity level, 2) frequency of movement and 3) intensity of involvement in an activity. Questionnaire items were developed from literature on children's after school activities and in conjunction with a workgroup of paediatric health care professionals, researchers working with young children, and parents of children in the relevant age group. The activities were chosen to represent activities in which 5- to 7-year-olds commonly engage during after-school hours. The aim was to create a questionnaire that could be answered in 1 to 2 minutes. Pilot testing revealed response time ranged from 0.5 to 3 minutes, with a shorter response time as respondents became increasingly familiar with the questions.

Parents record responses to diary questions on a Palm Pilot Z22 (Palm Inc., Sunnyvale, CA) loaded with Experience Sampling Program (ESP) software http://www.experience-sampling.org. For each diary entry, the Palm emits a signal that continues for 3 minutes before the device is switched off and the entry is marked as a non-response. Once the survey commences, a 3-minute lag of non-activity is allowed to accommodate interruptions before switching off. Any questions completed prior to the non-activity are saved.

##### Protocol

Parents are instructed in use of the Palm in a personal meeting with the researchers or through a step-by-step instruction sheet included with the Palm pilots when they are distributed to the parents. Parents are provided with a contact number, should they encounter any problems with the devices. On the morning of first day of data collection, researchers activate the software and place the devices in the participating children's schoolbags together with the accompanying information and written instructions. After the 4 days of data collection, parents return the Palm Pilot to their child's school. Data from the Palm Pilots are downloaded onto PCs using the ESP desktop software and stored as .txt files.

#### Videotaping

Digital hard drive video cameras (Sony DCR-SR65, http://www.sony.com.au) are used together with Bluetooth wireless microphones (ECH-HW1, http://www.sony.com.au) to capture social interactions and what the children are doing during recess. Children are videotaped for 15 minutes during recess by an unobtrusive camera person who does not interact in any way with the children. No videotaping occurs during bad weather when the children are indoors during recess; researchers also avoid taping children during lunch or snack time. The videos are transferred to computers using the accompanying software and stored as .mpg files. After conversion to .mov files, the resulting data are coded using Studio Code (v 4.2.4, http://www.studiocode.com). The coding scheme is developed specifically for this study and captures categories of play and non-play, as well as quantification of social interactions.

#### Children and Teachers' Perceptions of Competence and Social Acceptance

The Pictorial Scale of Perceived Competence and Social Acceptance for Young Children (PSPCSAYC) (Harter and Pike, 1984) [60] is included to assess two domains of competence (physical and academic) and two domains of acceptance (peer and maternal). Administration of the PSPCSAYC to children involves responding to four choices elicited by presenting paired pictorial items featuring children who are skilled or less skilled in a particular domain and asking who the child is most like. Further probing occurs to see if they see themselves as a lot like the child or just a little. The questions are devised to guard against generation of socially desirable responses.

The teacher rating scale involves a description of specific skills in each domain, with the exception of maternal acceptance. Teachers respond using a 4-point scale. The PSPCSAYC is widely used. For example, the original article describing the scale has 374 citations in Scopus (21 July, 2011).

#### Social Skills Improvement System - social skills

The Social Skills Improvement System Rating Scales (SSIS-RS) [61] are a revision of the widely used Social Skills Rating System (SSRS) [62]. For children below 8 years of age, parent and teacher reports of behaviours, using a 4-point scale, are used to assess Social Skills (i.e., communication, cooperation, assertion, responsibility, empathy, engagement, and self-control); and Problem Behaviours (i.e., externalizing, bullying, hyperactivity/inattention, internalizing, and autism spectrum).Teacher reports also includes an Academic Competence subscale. The SSIS-RS has been specifically designed for pre- and post-intervention assessment. Significant re-norming was conducted in the revision from the SSRS [62]. Protocols described in the SSIS-RS manual, such as ensuring the rater has known the child for at least 2 months, are followed. Parents of the children are provided with feedback on their child's score and in cases of extreme scores, are offered follow-up with the psychologist on this project. The completion time is 10 to 25 minutes per form. Compensation of teachers' time is offered by paying the schools for relief teaching staff. Scores are entered manually into the SSIS Assist software http://www.pearsonpsychcorp.com.au.

#### Anthropometry

Height and weight are measured using standard procedures. Height is measured to the nearest 0.1 cm using a portable stadiometer. Weight is measured to the nearest 0.1 kg using digital scales; children will wear school uniform (shoes removed).

#### Tolerance of Risk in Play Scale (TRiPS)

We are developing and testing the validity and reliability of data gathered with an instrument to examine adults' tolerances of risk during children's play. The scale is based on a Norwegian model of risky play [63]. We will collect data from 100 parents and teachers of children aged 3 to 13 years.

#### Qualitative data

Qualitative data in the form of audio recordings of risk reframing sessions, brief, written participant evaluations, and in-depth interviews with selected teacher and parent volunteers are gathered to understand some of the experiences and observations of the adults participating in the study.

#### Data analysis

Primary outcomes (activity levels) will be analysed using intention-to-treat principles. The effectiveness of the intervention for increasing activity, changing social skills and after school activities will be measured at both cluster and individual levels. Mixed-effects multi-level regression (STATA/IC 12, http://www.stata.com), taking clustering and repeated measurement of participants into account, will be used to examine net change from baseline values between groups. At the individual level, multivariate regression will be used to examine the contributions of activity level to changes in self-concept and social skills between groups. Secondary analyses will be conducted to explore subgroup effects (e.g., by sex). In addition, the contribution of potential confounders such as BMI will be explored.

Data from the audio recordings of the risk reframing sessions, parent interviews and field notes kept by project staff will be transcribed either in their entirety or as brief written reports. The resulting qualitative data will be converted to text to be analysed according to an adaptation of Charmaz's [64] approach to social analysis. Initial emergent themes will be identified and data coded for patterns and complexity regarding participants' experiences, actions, beliefs or relationships to risk, assumptions regarding risk, the larger process of which this action or belief is a part, and possible implications of such actions or beliefs for particular actors, institutional forms, parents, families, or children.

#### Process evaluation

During and after each round of data collection, the process is evaluated and the methods of data collection and communication are reassessed to monitor fidelity to the treatment. Teachers' and administrators' perceptions and attitudes are assessed by survey and informal interviews. Photos, videos and onsite observations of the school playgrounds are used to monitor and evaluate the progression of the use of unstructured materials.

## Discussion

Children's obesity, bullying and poor social skills represent a challenge to their mental and physical health. These three are often inter-related, but can be reversed. Many programs aiming at increasing physical activity have been implemented in the last decades, but most of them fail to have the potential to be adopted everywhere anywhere, due to structure, cost and lack of appeal to some children. This project is novel in investigating the benefits of free play on the playground for increasing physical activity and social skills in children and challenging adults to consider how their perceptions of risk may impact children's access to active free play. Due to its scope and potential to be adopted anywhere, if we have positive findings, this project has the prospect to influence policy around school-, and public playgrounds.

## Competing interests

The authors declare that they have no competing interests.

## Authors' contributions

ACB is the principal investigator, conceived of the study and led the study design and coordination and critically revised the manuscript. GN, PT, SW, LB, WS and AB are chief investigators and all contributed to the design of the study and critically revised the manuscript. LE is the project manager and drafted the manuscript. TL and JB contributed to the conceptualisation and design of the project. JR and AN contributed to the design of the project. GS and GJ contributed to fine-tuning of the methodology. All authors read and approved the manuscript.

## Pre-publication history

The pre-publication history for this paper can be accessed here:

http://www.biomedcentral.com/1471-2458/11/680/prepub
